# Validation and Clinical Utility of the Korean Version of the Obstetric Quality-of-Recovery Score (ObsQoR-11) Following Elective Cesarean Section: A Prospective Observational Cohort Study

**DOI:** 10.3390/diagnostics12020291

**Published:** 2022-01-24

**Authors:** RyungA Kang, Seungwon Lee, Eunkyung Lee, Yoon Jee Cho, Ji Seon Jeong, Soo Joo Choi, Mi Sook Gwak, Woo Seog Sim, Duk Kyung Kim, Justin Sangwook Ko

**Affiliations:** Department of Anesthesiology and Pain Medicine, Samsung Medical Center, Sungkyunkwan University School of Medicine, Seoul 06351, Korea; seungwon0209.lee@samsung.com (S.L.); eunk.lee@samsung.com (E.L.); yoonjee.cho@samsung.com (Y.J.C.); jiseon78.jeong@samsung.com (J.S.J.); sjoo.choi@samsung.com (S.J.C.); ms.gwak@samsung.com (M.S.G.); wooseog.sim@samsung.com (W.S.S.); dk68.kim@samsung.com (D.K.K.); jsko@skku.edu (J.S.K.)

**Keywords:** cesarean section, patient-reported outcome measures, quality of recovery, obstetric anesthesia

## Abstract

The Obstetric Quality of Recovery (ObsQoR-11) score is a new scoring tool that assesses maternal recovery after cesarean section (CS). We aimed to validate the translated Korean version of ObsQoR-11 (ObsQoR-11K) after elective CS. We validated ObsQoR-11K between March 2021 to August 2021. Validity (convergent, discriminant, and construct), reliability (Cronbach’s α, inter-item, split-half, and test-retest correlation), responsiveness, and clinical feasibility (recruitment rate and time for ObsQoR-11K completion) of ObsQoR-11K were evaluated. One hundred and twenty women completed the ObsQoR-11K 24 h after CS, and 24 women repeated it 25 h after CS. We found good convergent validity between the ObsQoR-11K score and the global health numerical rating scale (NRS) (ρ = 0.73 (95% CI 0.64 to 0.81); *p* < 0.001). The ObsQoR-11K score discriminated well between good (NRS ≥ 70 mm, *n* = 68, 69.6 ± 13.7) and poor recovery (NRS < 70 mm, *n* = 52, 50.6 ± 12.6, *p* < 0.001). The ObsQoR-11K score showed acceptable internal consistency (Cronbach’s α = 0.78), split-half reliability (0.89), intra-class correlation > 0.4, and no floor or ceiling effect. Of the participants, 100% completed the ObsQoR-11K and median (IQR) time for ObsQoR-11K completion was 81 s (66–97.5 s). ObsQoR-11K is a valid and reliable scoring tool for assessing maternal recovery after elective CS in Korean women.

## 1. Introduction

Cesarean section (CS) is one of the most frequently performed surgical procedures in obstetrics and gynecology [1]. Adequate assessment of the quality of recovery (QoR) after CS is necessary to improve maternal recovery [2,3]. However, QoR scoring tools for this obstetric population are limited. Several scoring tools, including QoR-40 [4], QoR-15, [5] EuroQol-5D [6], and Short Form Health Survey [7], have been used to measure maternal recovery after CS, but they have not been validated in obstetric settings. In addition, these scoring tools do not include items such as neonatal care, which is necessary for discharge. Moreover, since they were utilized for individuals under general anesthesia, they may not be suitable for the evaluation of mothers who mainly underwent regional anesthesia.

Recently, an obstetric-specific recovery scoring tool, the Obstetric Quality of Recovery (ObsQoR-11) score has been formulated and validated in patients undergoing elective CS [8] and non-elective CS [9]. Development studies on elective and non-elective CS patients suggested that ObsQoR-11 was a valid and reliable evaluation tool for CS [8]. However, the Korean version of ObsQoR-11 (ObsQoR-11K) has not been previously validated. Therefore, we conducted a prospective observational study validating ObsQoR-11K after elective CS and hypothesized that ObsQoR-11K would have similar psychometric characteristics in assessing the quality of postoperative recovery to the original English version.

## 2. Materials and Methods

This was a prospective observational study. After approval by the Institutional Review Board of Samsung Medical Center, Seoul, Korea (SMC 2020-12-101), this study was registered at the Clinical Research Information Service (CRIS, http://cris.nih.go.kr, identification number: KCT0005794, date of registration: 20 January 2021; date of last access: 30 September 2021; principal investigator’s name: RyungA Kang) and conducted from March 2021 to August 2021 at Samsung Medical Center, Seoul, Korea. Written informed consent was obtained from all women before surgery, and the study was conducted in accordance with the Declaration of Helsinki. All methods were performed according to the Strengthening the Reporting of Observational Studies in Epidemiology guidelines [10].

We enrolled 120 women who underwent elective CS under neuraxial anesthesia at ≥37 weeks of gestational age. Exclusion criteria were age under 19 years, patients under general anesthesia, inability to understand written Korean or the study protocol, unwillingness to participate, and history of severe psychological disorders that could affect evaluation.

We performed the following process to translate the original English version into Korean before the initiation of the study process. The translation process was accomplished based on recommendations [11] and previous validation studies [8,12]. First, two authors (R.K. and S.L.) translated the ObsQoR-11 into Korean based on the Korean version of the QoR-40 (QoR-40K) [13] and QoR-15 (QoR-15K) [12], which had been validated. Items 7, 8, and 9, which were not included in QoR-15K and QoR-40K, were translated by two authors each (item 7: I am able to move independently; item 8: I can hold my baby without assistance; item 9: I can feed/nurse my baby without assistance). Subsequently, the two translated versions were compared. Then, a consensus was reached through discussion. At a second step, one bilingual person (J.S.K.), who had completed university education in the USA, translated the Korean version back to English. Finally, three authors (R.K., S.L., and E.L.) compared the original questionnaire with the backward-translated questionnaire and assessed each item according to its degree of accordance using a 7-point scale (1, no accordance; 7, perfect accordance). Items on a scale of 5 to 7 points were accepted, and items that did not meet these criteria were reviewed. The ObsQoR-11K used in this study is shown in Figure S1.

Informed consent was obtained the day before surgery. One of two investigators (S.L. and E.L.) who were not involved in clinical care interviewed the participants and asked the questionnaire. Baseline demographic and clinical data (age, body mass index, parity, gestation, cause of elective CS, underlying disease, previous CS history, American Society of Anesthesiologists (ASA) physical status classification) were collected at the time of enrolment. Women were approached while in the postnatal ward 24 h after CS and asked to complete the ObsQoR-11K questionnaire and to choose a point in the 100-mm numeric rating scale (NRS) to evaluate global health status. Women rated each recovery item using an 11-point numerical Likert scale (0 = strongly negative; 10 = strongly positive). Global health status was measured using NRS indicated by a 100 mm line (0 = ‘worst imaginable health status’ and ‘sad’ face; 100 = ‘best imaginable health status’ and ‘happy’ face). Twenty-four (20%) participants were chosen randomly and asked to repeat the ObsQoR-11K 1 h later (i.e., 25 h) as a measure of test-retest reliability. We also collected data on the duration of surgery, duration of anesthesia, estimated blood loss, duration of post-anesthetic recovery unit (PACU) stay, maximum pain score while in PACU, spinal level at admission and discharge from PACU, pre- or postoperative levels and differences in hemoglobin concentration, and length of hospital stay. All ObsQoR-11K questionnaires were completed under the guidance of the investigator.

All women were instructed to fast for 6 h before surgery and allowed to drink clear fluid until 2 h before surgery. At our institution, a combined spinal-epidural technique using two separate needles with the separate needle technique was a standard clinical anesthesia guideline for elective CS [14,15]. Upon the arrival of patients in the operating room, standard ASA monitoring was applied. With the patient in the right lateral position, a 19-gauge epidural catheter was inserted via a 17-gauge Tuohy needle (FlexTip Plus^®^; Arrow International, Inc., Reading, PA, USA) at the L2-3 intervertebral space using a midline approach with the loss-of-resistance to air technique and fixed at 10–11 cm into the skin. Spinal tapping was performed at the L3-4 interspace with a 25-gauge Whitacre needle, and 8 mg 0.5% hyperbaric bupivacaine and 20 µg fentanyl were administered. An epidural injection of 10 mL 0.2% ropivacaine was administered thereafter. The success of anesthesia was assessed bilaterally using a cold alcohol swab and pin-prick test, and if the sensory dermatome of T4 was confirmed, surgery was started.

All women received a standardized postoperative supplemental analgesic regimen. Patient-controlled epidural analgesia (PCEA) with 1000 μg fentanyl and 40 mL 0.75% ropivacaine mixed with 210 mL 0.9% saline was initiated in PACU at the first complaint of pain (NRS > 1/10) and programmed to deliver a background infusion of 4 mL/h with 2 mL bolus and a 15-min lockout interval until postoperative day (POD) 2. After the epidural catheter was removed, 100 mg oral aceclofenac (Airtal^®^, Daewoong Pharmacy, Seoul, Korea) was prescribed twice a day for PODs 2 and 3. Patients with breakthrough pain (NRS > 4/10) were administered 100 mg ketoprofen (ketoprofen 100 mg/2 mL, Bukwang Pharmacy, Seoul, Korea) intramuscularly up to three times from POD 0 to POD 3. Postoperative nausea or vomiting was treated with 0.3 mg intravenous ramosetron (Naseron, Boryung Pharmacy, Seoul, Korea), if needed. Women were encouraged to move 6 h after spinal anesthesia, and the urinary catheter was removed 24 h after surgery.

Previous studies described that power analysis is not reliable for calculating the sample size for correlation analysis [9,16]. Therefore, the sample size of this study was guided by previous studies [8,9] assuming a 20% dropout rate. We planned to recruit 120 women who were admitted for elective CS under regional anesthesia in our institution.

Normal distribution of continuous variables was tested using the Shapiro–Wilk test. Normally distributed continuous variables were presented as the mean ± standard deviations (SDs) and compared using a two-sample Student’s t-test. When the distribution was not normal, median (interquartile ranges (IQRs)) was presented and the groups were compared using a Mann–Whitney U-test between two independent groups or a Wilcoxon signed-rank test for paired data. Categorical variables are presented as numbers (percentages) and were compared between groups using the chi-square test or Fisher’s exact test, according to their expected counts. Correlations were measured using Spearman’s correlation coefficient (ρ).

Validity, reliability, responsiveness, and clinical acceptability of ObsQoR-11K was evaluated based on the following analyses based on previous publications [8,9]. First, we evaluated the convergent, discriminant, and construct validity of ObsQoR-11K. To evaluate convergent validity, ObsQoR-11K scores at 24 h and 25 h after surgery were correlated with the 100-mm NRS assessment of global health status at the same time. For discriminant validity, a comparison was made between the 24 h ObsQoR-11K of women who had a “good” or “poor” postoperative recovery, defined by global NRS scores ≥70 vs. <70 mm at 24 h. For construct validity, the correlation between the ObsQoR-11K score and length of stay, age, body mass index, parity, gestational age, gestation (singleton vs. multiple), previous CS history, duration of surgery, estimated blood loss during surgery, pre- and postoperative hemoglobin concentration, and changes in hemoglobin concentrations were estimated. These outcomes were selected on the basis of previous studies [8,9]. Second, reliability was assessed by internal consistency, inter-item correlation tests, split-half reliability, and test-retest reliability. Internal consistency was measured using Cronbach’s α, and test-retest reliability was measured using the intraclass correlation coefficient (ICC). Floor and ceiling effects were evaluated based on whether <15% of respondents achieved either the lowest (0) or highest possible score (110). Third, acceptability and clinical feasibility of ObsQoR-11K were evaluated using the patient recruitment rate, as determined by the percentage of women who agreed to complete the scoring tool, by successful completion rate based on the recording of the number of correct ObsQoR-11K forms without missing data, and by the time required to complete the ObsQoR-11K, measured and recorded by the investigator.

Data analysis was performed with SAS version 9.4 (SAS Institute Inc. Cary, NC, USA) or R version 4.0.3 (R Foundation for Statistical Computing, Vienna, Austria). Differences were considered statistically significant when the *p*-value was < 0.05.

## 3. Results

**Figure 1:**
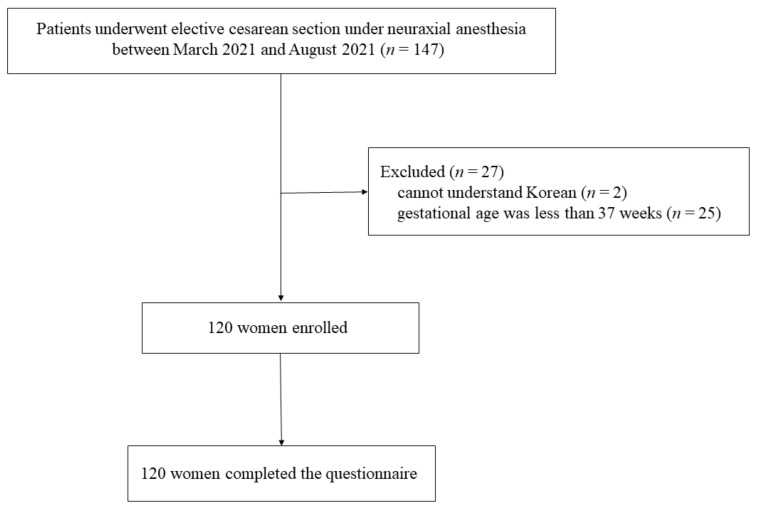
Flow diagram of this study.

From March 2021 to August 2021, 147 women scheduled for elective CS were assessed for eligibility. Of these, 27 women were excluded prior to enrolment because they did not meet the inclusion criteria (Figure 1).

A total of 120 women were recruited (recruitment rate: 100%) and completed the ObsQoR-11K questionnaire 24 h after CS (completion rate: 100%). Of these, 24 women (20%) repeated the ObsQoR-11K questionnaire 25 h after CS. Clinical characteristics of the women are presented in Table 1.

Individual item correlation to the global health NRS score is shown in Table 2.

The ObsQoR-11K scores 24 h after CS were significantly correlated with global health NRS scores at 24 h (ρ = 0.73 (95% CI 0.64 to 0.81), *p* < 0.001). ObsQoR-11K scores differed significantly between two groups divided according to their global health NRS score; “good” (*n* = 68, 69.6 ± 13.7) vs. “poor” postoperative recovery (*n* = 52, 50.6 ± 12.6, *p* < 0.001). There was no significant correlation between ObsQoR-11K scores and clinical characteristics (Table 3).

Inter-item Cronbach’s α was 0.78 and split-half reliability was 0.89. The inter-item correlation matrix for ObsQoR-11K is presented in Table 4.

Inter-item correlations were mostly ρ > 0.15, which indicates good consistency. The test-retest ICC for total ObsQoR-11K scores was 0.76 and ρ > 0.46 (range 0.46–0.92) for all items, suggesting adequate reproducibility and reliability (Table 5). There were no floor or ceiling effects. The median (IQR) time required to complete the ObsQoR-11K questionnaire was 81 s (66–98), with a range from 38 to 309 s.

## 4. Discussion

Our study demonstrates that ObsQoR-11K has excellent validity, reliability, clinical acceptability, and feasibility in patients undergoing elective CS. Specifically, ObsQoR-11K showed a strong correlation with global health NRS scores 24 h after CS (ρ = 0.73). Internal consistency, measured by Cronbach’s α and split-half reliability, remained above the recommended levels (0.7–0.9) [17]. These results were consistent with the original English version of ObsQoR-11 [8,9]. Therefore, the ObsQoR-11K is an acceptable method for assessing postoperative recovery in the acute postoperative period for Korean mothers.

Our results showed the following characteristics compared with the original version of the ObsQoR-11. First, in our study, no significant correlation was found between the ObsQoR-11K scores and clinical characteristics. In the original version of ObsQoR-11, there was a weak negative correlation between the length of hospital stay and ObsQoR-11 (r = −0.24; *p* = 0.02), and a weak positive correlation between parity and ObsQoR-11 (r = 0.24; *p* = 0.02) in patients who underwent non-elective CS [9]. This difference may be due to differences in the clinical environment and patient characteristics. In our study, most women were discharged on POD 3 (i.e., four nights stay) to follow the institutional clinical guidelines. However, the length of stay in the previous study was shorter than in our study (i.e., median (IQR) length of stay was 3 (2–4) nights) [9]. Patient characteristics also differed; in our study, 58.3% of women were nulliparous, whereas, in the previous study, 72% of women were nulliparous. Second, ObsQoR-11K scores (mean 67) for women with good postoperative recovery were lower than those in the original version of previous studies involving elective CS (median 100) and non-elective CS (median 97). This difference may be associated with the prolonged urinary catheterization in our study. At our institution, 27 women (23%) had a urinary catheter when asked the ObsQoR-11K questionnaire, to conform with institutional obstetric guidelines to accurately calculate inputs and outputs. After 24 h, the urinary catheter was removed, and women were encouraged to ambulate the ward. Prolonged urinary catheterization can lead to bladder irritation and vague abdominal discomfort [18]. Therefore, these differences in the clinical environment may account for the lower good recovery scores observed in our study compared to previous studies.

In this study, some patients were confused with the change in the scale definition parts I and II, which was also pointed out in previous studies [8,9]. Thus, the investigators had to help those patients to clearly understand the scale. In addition, because more than half of the mothers were in their first childbirth, it was difficult to obtain high scores on items 8 (hold baby) and 9 (nurse baby). However, the acceptability and clinical feasibility of ObsQoR-11K were high. The time required to complete ObsQoR-11K was 81 s and the participant’s refusal rate was zero. Therefore, ObsQoR-11K was easy quick to use and this is an advantage of this scoring tool.

This study had several limitations. First, all interviews and interactions with participants were conducted at a single tertiary hospital in Korea. Therefore, the clinical conditions (surgical conditions, anesthesia, postoperative management, and analgesic) appeared to be very homogeneous for all women enrolled in this study, which may have contributed to the good results of the study. Further evaluation and validation are required in hospitals that serve patients with different characteristics to extend ObsQoR-11K application during the emergency CS or vaginal delivery. Second, ObsQoR-11K was only measured on POD 1 without further serial assessment. As a patient’s mental and physical status undergoes rapid changes during the acute postoperative phase, changes in ObsQoR-11K throughout the postoperative hospital stay may have more profound clinical implications than a single measurement.

## 5. Conclusions

In conclusion, the Korean version of the ObsQoR-11K has acceptable validity, reliability, and feasibility for assessing the quality of recovery after CS despite cultural differences. It is expected that ObsQoR-11K will be a useful tool for evaluating the quality of recovery of Korean women in the future.

## Figures and Tables

**Table 1:** Demographic and clinical characteristics of women completing the ObsQoR-11K questionnaire.

Parameter	*n* = 120
Age (years)	
Mean ± SD	35.5 ± 4.1
Range	20–48
Body mass index	
Median (IQR)	26.1 [23.7–28.0]
Range	19.3–46.7
Parity	
0	70 (58.3)
1	45 (37.5)
2	3 (2.5)
3	2 (1.7)
Gestation	
Single	108 (90.0)
Multiple	12 (10.0)
Length of hospital stay (days)	
Median (IQR)	4 [4–4]
Range	4-11
Pre-existing medical conditions, *n* (%)	
Respiratory	2 (1.7)
Cardiovascular	11 (9.2)
Neurological	1 (0.8)
Endocrine	10 (8.3)
Hematological	14 (11.7)
Musculoskeletal	1 (0.8)
Psychiatric	0 (0.0)
Other	14 (11.7)
Obstetric indication for CS, *n* (%)	
Maternal choice	31 (25.8)
Previous CS	39 (32.5)
Multiple gestation	12 (10.0)
Cephalopelvic disproportion	2 (1.7)
Placenta previa	8 (6.7)
Other abnormal position	1 (0.8)
Breech	14 (11.7)
Others *	13 (10.8)
Previous CS, n (%)	
Yes	40 (33.3)
No	80 (66.7)

Data are presented with mean ± standard deviation (SDs), median (interquartile ranges), or numbers (percentages). * Others included 7 women who had a history of the previous myomectomy, 1 newborn expected large for gestational age, 1 woman suspected of marginal placenta abruption, 1 woman suspected of the low lying placenta, 2 women who had birth canal obstruction due to huge myoma or cyst, and the last one had asthma with dyspnea on exertion. CS: cesarean section, DM: diabetes mellitus.

**Table 2:** Summary of correlations of ObsQoR-11K items to global health numerical rating scale (NRS).

ObsQoR-11K Item	Correlation to Global Health NRS Score * Spearman r (95% CI)	*p*-Value
Moderate pain	0.53 (0.38 to 0.65)	<0.001
Severe pain	0.38 (0.19 to 0.56)	<0.001
Nausea or vomiting	0.28 (0.10 to 0.45)	0.002
Dizzy	0.31 (0.14 to 0.48)	0.001
Shivering	0.30 (0.14 to 0.46)	0.001
Comfortable	0.62 (0.48 to 0.74)	<0.001
Mobilize independently	0.53 (0.39 to 0.66)	<0.001
Able to hold baby	0.35 (0.18 to 0.50)	<0.001
Able to nurse/feed baby	0.34 (0.17 to 0.50)	<0.001
Able to take care of personal hygiene	0.50 (0.34 to 0.64)	<0.001
Feeling in control	0.27 (0.09 to 0.43)	0.003

* Global health NRS represented as a 100 mm line, marked at each end with anchors “worst imaginable health state” to “best imaginable health state” and with “sad” or “happy” stylized representations of faces. CI, confidence interval.

**Table 3:** Summary of correlations of clinical characteristics to ObsQoR-11K score.

Clinical Characteristic	Correlation to ObsQoR-11K Spearman r (95% CI)	*p*-Value
Length of stay	−0.01 (−0.17 to 0.16)	0.934
Parity	0.01 (−0.17 to 0.21)	0.887
Gestational age	0.13 (−0.04 to 0.29)	0.151
Gestation (singleton vs twins)	−0.05 (−0.22 to 0.12)	0.602
Maternal age	−0.12 (−0.28 to 0.07)	0.198
BMI	−0.03 (−0.21 to 0.16)	0.763
Category of CS	−0.12 (−0.30 to 0.08)	0.204
Previous CS	0.07 (−0.11 to 0.26)	0.473
Duration of surgery	0.09 (−0.09 to 0.27)	0.343
Blood loss	−0.06 (−0.24 to 0.14)	0.551
Preoperative Hb	0.03 (−0.16 to 0.22)	0.770
Postoperative Hb	0.08 (−0.09 to 0.26)	0.375

BMI: body mass index, CS: cesarean section, Hb: hemoglobin concentration.

**Table 4:** Inter-item correlation matrix for ObsQoR-11K.

ObsQoR-11K Item Number *	Global Health NRS ^¥^	Total ObsQoR-11K Score	1	2	3	4	5	6	7	8	9	10	11
1	0.53	0.65	-										
2	0.38	0.56	0.55	-									
3	0.28	0.43	0.26	0.20	-								
4	0.31	0.41	0.14	0.18	0.52	-							
5	0.30	0.39	0.20	0.09	0.31	0.21	-						
6	0.62	0.67	0.51	0.52	0.28	0.15	0.16	-					
7	0.53	0.62	0.28	0.29	0.08	0.08	0.09	0.54	-				
8	0.35	0.62	0.26	0.10	−0.01	0.05	0.18	0.23	0.52	-			
9	0.34	0.60	0.18	0.10	0.04	0.05	0.17	0.20	0.40	0.79	-		
10	0.50	0.65	0.34	0.27	0.06	0.12	0.09	0.29	0.54	0.63	0.66	-	
11	0.27	0.38	0.09	0.27	0.34	0.33	0.23	0.28	0.12	0.05	0.09	0.04	-

* Obstetric Quality of Recovery score items (1 = moderate pain; 2 = severe pain; 3 = nausea or vomiting; 4 = feeling dizzy; 5 = shivering; 6 = have been comfortable; 7 = able to mobilize independently; 8 = can hold my baby without assistance; 9 = can feed/nurse baby without assistance; 10 = can look after personal hygiene/toilet; 11 = feeling in control). ^¥^ Global health NRS represented as a 100 mm line, marked at each end with anchors “worst imaginable health state”; to “best imaginable health state” and with “sad” or “happy” stylized representations of faces.

**Table 5:** Intraclass correlation coefficient (ICC) for ObsQoR-11K items.

ObsQoR-11K Item	ICC
Moderate pain	0.62
Severe pain	0.54
Nausea or vomiting	0.78
Dizzy	0.89
Shivering	0.89
Comfortable	0.46
Mobilize independently	0.61
Able to hold baby	0.56
Able to nurse/feed baby	0.52
Able to take care of personal hygiene	0.83
Feeling in control	0.92
Total ObsQoR-11K score	0.76

Global health NRS represented as a 100mm line, marked at each end with anchors “worst imaginable health state” to “best imaginable health state” and with “sad” or “happy” stylized representations of faces.

## Data Availability

The data presented in the study are available on request from the corresponding author.

## Supplementary Materials

The following supporting information can be downloaded at: https://www.mdpi.com/article/10.3390/diagnostics12020291/s1, Figure S1: The ObsQoR-11K used in this study.
