# Supplementary figures and images for: Validation and Clinical Utility of the Korean Version of the Obstetric Quality-of-Recovery Score (ObsQoR-11) Following Elective Cesarean Section: A Prospective Observational Cohort Study
Source: Diagnostics (Basel). 2022 Jan 24;12(2):291. doi: 10.3390/diagnostics12020291 (PMC8871019; doi:10.3390/diagnostics12020291)

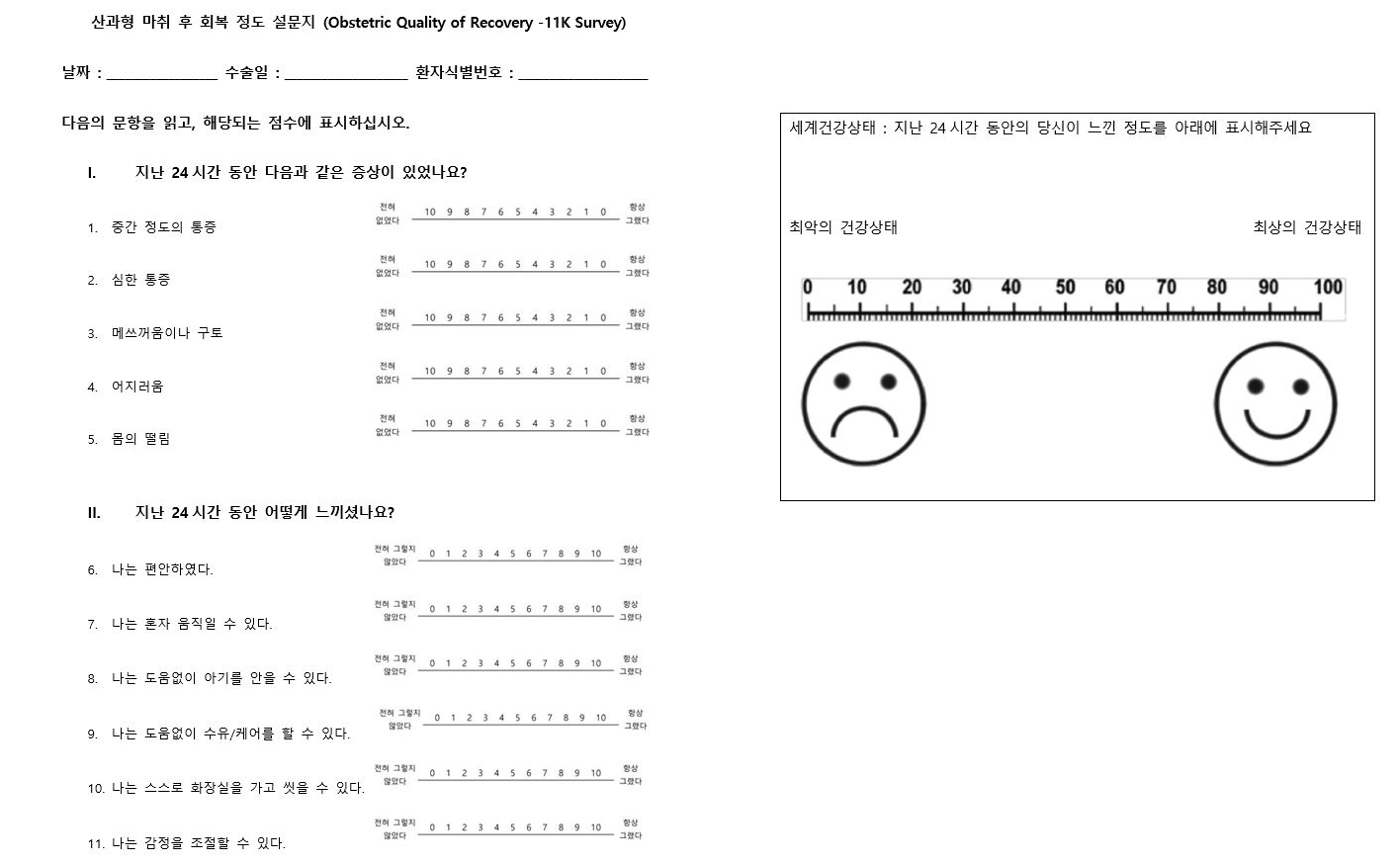

Supplement: Supplementary file 1 [file diagnostics-12-00291-s001.zip › diagnostics-1547590-supplementary.tiff]
